# Statistical Details of Four Years Experience in Respect to the Form of Amaurosis Supposed to Be Due to Tobacco

**Published:** 1872-10

**Authors:** Jonathan Hutchinson

**Affiliations:** From the Royal London Ophthalmic Hospital Reports


*Statistical Details of Four Years' Experience in Respect to the Form of Amaurosis Supposed to be Due to Tobacco.* By Jonathan Hutchinson, F.R.C.S. From the Royal London Ophthalmic Hospital Reports, vol. VII, part II, p. 169.

In the year 1864 I published, in the first volume of the "London Hospital Reports," under the title of "Clinical Data Respecting Cerebral Amaurosis," etc., a report on cases supposed to be connected with tobacco. I then gave all the cases of which I had notes which appeared to resemble the form due to smoking, including a considerable number in which it was quite certain that the habit referred to was not the cause. After this date, I carefully kept notes of every case bearing upon the subject which came under my notice, and in 1867 the Medico-Chirurgical Society did me the honor to publish in their Transactions, a statistical summary of my experience during the three intervening years. The cases included in that (second) report had been much more carefully selected, and from it several classes, which I had deemed it necessary to mention in my first, were wholly excluded. My recognition of the peculiarities of tobacco cases had also become somewhat more accurate. I now have to offer to the reader a third series of cases of the same kind, including most that came under my notice during the four years, 1867, 8, 9, and 70. I regret that I cannot say that during this period I have kept notes of quite all that have been under my care, more especially during the year 1868; but those which have been omitted, have been left out simply from want of time to take notes, not from any principle of selection. Each of my three several reports presents a fair picture of my experience in respect to all cases which could be considered similar to those due to smoking. Each includes a few in which I think it very doubtful whether or not smoking was the real cause. I have been most anxious to give to the reader a fair opportunity of judging for himself as to the clinical features which this form of amaurosis presents in English practice, and of forming his own opinion as to whether the indictment against tobacco is a just one.

Any one who may take the trouble to compare the cases given in these three reports, will be struck by their similarity, and by the remarkable confirmation which the two later papers give to the conclusions of the first. A very great disproportion in the numbers of the two sexes who become the subjects of "idiopathic amaurosis," was one of the facts brought out (for the first time) by my first report. In it the numbers were 37 men and 3 women, and in the second they were 34 men and 5 women. In the present one they will be found to be 28 men and only one woman. There can, therefore, be no reasonable doubt that we have to deal with a formidable type of symmetrical amaurosis which is almost exclusively confined to the male sex. For myself, I may briefly avow that I have scrupulously investigated other possible causes, and that I feel no hesitation in believing that in most of these cases tobacco is the real one.

The facts as regards the one woman whose case is included in the present series, are extremely interesting. Her case in every feature resembled those which in males I attribute to smoking, and upon this point I had made special comment to those who were attending my class. After I had done so, she informed me that one of her sons had formerly been my patient, and that a nephew had been under the care of Mr. Hulke. I referred to my notes, and Mr. Hulke was kind enough also to refer to his, and we each found that the cases had been diagnosed as tobacco amaurosis. In each, the patient was a young man and a smoker. Now the lesson of these facts seems to me to support the opinion I have long held, that when tobacco causes blindness it does so in virtue of an idiosyncrasy. It is by no means improbable that such idiosyncrasy will be found occasionally in several members of the same family, and further, that it may involve liability to suffer from other influences besides tobacco smoking. Thus, then, we have two young men, cousins, attacked by amaurosis at an early age in virtue of an idiosyncrasy rendering them liable to special poisoning from tobacco; and we have a woman, who had never smoked, the mother of one and aunt of the other, at a much later period of life becoming the subject of a similar form of nerve disorder in virtue of the same peculiarity of diathesis acted upon by a different exciting cause. In her, not improbably the exciting cause was the disturbance consequent upon the cessation of menstruation.

Holding the opinion that there must be some pre-existing peculiarity in the nervous systems of those who become the subjects of tobacco amaurosis, I have been very anxious to discover, if possible, whether it reveals itself by any other signs. The only points in this direction to which I feel at present inclined to attach any importance are the following. Not unfrequently it will be found that these patients have had unusual difficulty in learning to smoke, and have throughout life displayed special susceptibility to its influence, and that also they have often been, beyond the average, liable to suffer from sea sickness. In the tabular statement some information will in many cases be found given on these points. My attention to the matter of sea sickness was first given in consequence of the statements of some patients who were sailors, and who specially referred to it.

We have, during the last few years, made the observation that those who suffer are, almost invariably, smokers of *shag*, the most deleterious form of tobacco. As regards the results from disuse of tobacco my impression is strong, that almost invariably when the disuse is real and complete, the state of vision improves. I cannot exaggerate the expression of my conviction as to the duty of urging immediate and complete abstinence in the early stages of this most serious malady. Many of my patients came too late, having continued to smoke until the disease was far advanced. I have never seen a case in the early stage in which the disease went on to blindness if the patient had strength of will to give up the habit. In a large majority of those in whom, according to the patient's account, the habit was wholly abandoned, I had reason to suspect that it was only reduced. My experience on this point fully confirms that of Dr. Mackenzie, that there are those "who would rather smoke than see." It is very few, however, who have the honesty to admit their inability to give up the habit, and hence a very annoying source of fallacy in our inferences as to the effect of treatment.

It will be seen that in a few cases in the table, the amaurosis set in rather suddenly and advanced rapidly. This fact I always take as a strong one against the diagnosis of tobacco amaurosis, and in favor of that of neuritis. These cases ought, therefore, very probably to have been excluded from the list. As, however, the diagnosis is uncertain, I have preferred to let them stand. It so happens that some of these are the very ones in which no improvement resulted from the disuse of tobacco.

I have to thank my friends, Mr. Waren Tay and Mr. Nettleship, for much help in recording the notes of the cases and in compiling this report:

| Number. Date. Reference | Name. Occupation. State of Health. | Age. Duration of Amaurosis. | Use of Tobacco and Alcohol. | State of Eyes. | Other Symptoms. | Progress and Remarks. |
|---|---|---|---|---|---|---|
| 1. 1867, January 3. C., p. 94. | John Brewer, bricklayer. Has had good health. | 22. 18 months. | Has smoked from a quarter to half an ounce of tobacco daily for five years and a half. | Left eye worse than right. Right sees 20 J. Pupils large, and the right one sluggish. Each disc pale, especially at its outer part. | Has been troubled with nocturnal emissions. Denies syphilis. Gradual failure of sight. | 1867, *March* 7. Discs still paler, veins large, arteries small. Vision as before. He now says that he had not smoked for two years before the sight failed. |
| 2. 1867, March 11. C., p. 157. | Samuel Park, shoemaker. Pale, and weak looking. | 35. About 2½ to 3 years. | Has smoked for thirteen years; at one time he smoked an ounce of tobacco daily; has left off smoking several times, and resumed it again. Has usually been "worse for liquor" once every few months, but does not consider himself a great drinker. | Vision unequal in the two eyes; right, 1 J. and 20–40; left, 16 J. and 20–200. No material difference with glasses. Discs of a redder color than natural, but otherwise normal. Pupils act well. | History of weakness of sight (probably failure of accommodation) in boyhood. Smoking makes him nervous if he exceeds his usual amount. Sight has varied a good deal within the last two or three years, being, as he believes, worse when he leaves off smoking. | Has several times given up smoking for months together, because it made him nervous (made his hands shake, etc.), but has found that his sight always gets *worse* when he is not smoking. He is convinced that smoking is good for his sight; he gave it up only because it made him nervous. |

| Number.<br>Date.<br>Reference. | Name.<br>Occupation.<br>State<br>of Health. | Age.<br>Duration<br>of<br>Amaurosis. | Use of Tobacco<br>and Alcohol. | State of Eyes. | Other Symptoms. | Progress and<br>Remarks. |
|---|---|---|---|---|---|---|
| 3.<br>1868. | A woman. | 43.<br>6 months. | Had never smoked. Strictly moderate in stimulants. | White atrophy in both. | None of any moment. | The symptoms had come on gradually, just as in the tobacco cases in men. |
| 4.<br>1868,<br>March 1.<br>C., p. 259. | Moses Garcia, cab-driver. Looks strong and healthy, though sallow. | 53.<br>9 months. | Has smoked from half an ounce to an ounce daily. Has drunk moderately. | White atrophy of both discs. | At the time when his sight began to fail, his general health was bad; he was nervous and weak, troubled with sleeplessness, diminution of sexual appetite, and flatulence. Was obliged to give up his work, and was three months in the Jews' Hospital for dyspeptic symptoms. Has headache across the forehead. Has had gonorrhœa and chancres many times. No wasting of testes. | The failure of sight coincided in time with general failure of health. He had indulged his sexual appetite very freely. |

| | | | | | | |
|---|---|---|---|---|---|---|
| 5.<br>1869,<br>July 8.<br>C., p. 318. | John Thompson, bailiff, in comfortable circumstances. Florid and robust. Hair, sandy-grey. | 60.<br><br>9 months. | Has smoked for twenty years; for several years only a little; for five or six years past half an ounce a day. Has drunk alcoholic liquors constantly, but in moderate quantity. | Can see only 20 J. Discs white. | Has been very nervous for several years, especially after smoking in the morning. Many years ago suffered from "lumbago and sciatica," which laid him up for more than a year on and off. Gradual failure of sight. | 1869, *Sept.* 2. Has not smoked at all since he came here. His sight has got worse; cannot now count fingers. Discs both equally and universally white; veins and arteries somewhat diminished.<br>*Sept.* 23. Can barely find the position of the window. Pupils contracted; they do not dilate in the dark, but dilate moderately with atropine. |
| 6.<br>1869,<br>Sept. 23.<br>C., p. 324. | Wm. Billingham. | 35.<br><br>6 weeks. | Has smoked at least half an ounce daily. Has drunk moderately. | Vision 20–100 and 12 J. Discs pale at the outer halves; sheath at outer part very distinct, and a little disturbance of choroid. Inner margins indistinct, apparently from tendency to formation of crescents. | Has often noticed a nervousness from smoking. Gradual failure of sight. | |

| Number. Date. Reference. | Name. Occupation. State of Health. | Age. Duration of Amaurosis. | Use of Tobacco and Alcohol. | State of Eyes. | Other Symptoms. | Progress and Remarks. |
|---|---|---|---|---|---|---|
| 7. 1869, July 12. C., p. 327. | John Smith, dray-man. Dark complexion, and healthy. | 45. 3 months. | Has smoked "as much as he could get," chiefly *shag*. Has drunk moderately. | Vision 20–100 and 14 to 16 J. Discs decidedly whiter at outer than at inner halves; inner halves look slightly swollen. | Gradual failure. | 1869, *Sept.* 2. Vision 20–50 and 14 J. Considers his sight much better. Has left off smoking entirely. 1870, *June* 30. Vision 20–40 & 8 J. *Oct.* 13. Just sees letters of 6 J. *Dec.* 15. Just sees letters of 4 J. |
| 8. 1869, October 7. C., p. 337. | Thomas Willis, has been a soldier. | 42. 2 months. | Has smoked half an ounce daily for twenty years. Has not been in the habit of drinking while he smoked, and for two years has not drunk at all. | Vision 20–200 and 16 J. Discs ill-margined, choroid thin, outer half of each disc decidedly pale; veins full, and arteries not diminished. | Gradual failure. | 1869, *Nov.* 4. Vision 20–100 and 14 J. |
| 9. 1869, October (about). C., p. 339. | James Morris, engineer (light outdoor work); a Scotchman. Health good. | 40. 2 months. | Smokes an ounce daily of *pig-tail* tobacco. Drinks several glasses of brandy and some beer daily. | Commencing atrophy of discs. | Gradual failure. Is rather nervous; says he has noticed no ill effects from smoking except occasional "heart-burn." | |

| | | | | | | |
|---|---|---|---|---|---|---|
| 10. 1869, July 5. C., p. 312. | Henry Cartwright, steward on board ship. | 35. 2 months. | Has smoked half an ounce daily for twenty years. Has drunk a good deal. | Vision 19 J., and no letters in the distance. Discs decidedly paler at outer than inner halves. | Gradual failure; "is always in a fog." Has had no special inconvenience from smoking. | |
| 11. 1870, Feb. 3. C., p. 366. | Samuel Barnard, stableman. Square, and rather sallow. | 61. 12 months. | Has smoked "moderately" forty years; generally *shag* tobacco. Has been steady, and has drunk chiefly beer. | Vision, right eye cannot quite tell letters of 20 J. Left eye sees 14 J. Discs, right uniformly white; left paler at its outer than at its inner half. | Gradual failure; the right eye began to fail before the left, and is in a more advanced state of atrophy. | Is rather deaf; has been so for a year. 1870, *March* 15. Just the same. *Dec.* 15. Sees 10 J. Has not smoked at all. |
| 12. 1870, Jan. 10. C., p. 354. | R. J. Littleboy, sailor. | 55. 2 months. | Has smoked half an ounce daily for thirty years, chiefly *shag and cavendish* tobacco. Has been a great drinker, half a pint of spirits and two or three pints of beer daily, or more. | Vision 20–70. Discs pale at the outer halves. | None mentioned. | Has never been seasick. No difficulty in learning to smoke. 1870, *Mar.* 3. Vision 20–50, and 6 J. (with + 20). *Dec.* 15. His wife reports that he can see to read the newspaper. |
| 13. 1870, Feb. 21. C., p. 379. | George Jones. | 30. 18 months. | Has smoked nearly half an ounce daily for six years, of *shag* tobacco. Has drunk a good deal of gin and rum, but not generally when he smoked. | Vision 20–200 barely, and just sees 10 J. Discs, remarkably pale at the outer halves; vessels not much diminished. | Gradual failure. Had no difficulty in learning to smoke; is not sick when he goes on the sea. Memory has failed of late. | |

| Number. Date. Reference. | Name. Occupation. State of Health. | Age. Duration of Amaurosis. | Use of Tobacco and Alcohol. | State of Eyes. | Other Symptoms. | Progress and Remarks. |
|---|---|---|---|---|---|---|
| 14. 1870, March 24. F., p. 4. | Thomas Allen. | 53. 6 months. | Has used half an ounce daily, partly smoking, partly chewing, for more than thirty years, *shag* tobacco. Drinks porter pretty freely at times; does not drink while smoking. | Vision 20–200, and 16 J. | Gradual failure; it began as a fog. | 1870, *April* 11. Vision 20–70, and 16 J. |
| 15. 1870, April 11. F., p. 25. | Joseph Bryant, engineer. Strong, healthy man. | 56. 4 months. | Has smoked for forty years; half an ounce daily for last five years, *shag* tobacco. Formerly drunk five or six pints of beer daily; for several years past has drunk only half a pint, and occasionally a little rum. | Vision right eye 20–200, and 20 J., increased to 10 J. with glasses. Left eye lost many years ago after an injury by a chip of iron. Disc in right, blue white. | Gradual failure; no pain. | Sight was excellent in right eye before the recent attack. 1870, *Oct.* 31. Says the sight is worse; sees 5–200, and barely 10 J. with glasses. He wants to get compensation for an accident which has happened since he came before, and has, he says, made his sight worse. |

| | | | | | | |
|---|---|---|---|---|---|---|
| 16. 1870, May 5. F., p. 31. | Henry Lock, chair-maker. Pale, but in good health. | Not noted. 6 months. | Has smoked half an ounce daily for sixteen years, *shag* tobacco. Has usually taken two pints of beer daily; has never been intemperate. | Vision 20–100, and 10 J. Discs seem slightly swollen and indistinct; the outer half of each is whiter than the inner half; this difference is less marked in the right. | Gradual failure. Is rather deaf, especially on right side; has been so for several years. | 1870, *August* 25. Vision 2 J.; has improved notably. Has not quite left off smoking. Has taken nothing but iron, and used a little astringent lotion for the eyes. |
| 17. 1870, May 16. F., p. 34. | John Thornton. Spare, healthy, and vigorous; face rather florid. | 60. 7 weeks. | Has smoked (sometimes chewed) half an ounce daily for twenty years, *shag* tobacco. Drinks stout often when smoking, but considers himself temperate. | Vision 20–100, and 14 to 16 J. Discs pale, especially at outer halves; arteries not diminished. | Says the sight failed rather suddenly, & did not get much worse afterwards. History of occasional dimness for several years, probably due to weakness of ciliary muscle. Has been to sea a good deal, and is always sick for the first two or three days. | |
| 18. 1870, May 19. F., p. 39. | Frederick Graham, at present in a workhouse. | 48. 5 weeks. | Has smoked half an ounce daily for twenty years, of *shag* tobacco. | Vision 20 J.; nothing in the distance. Discs decidedly whiter at the outer than at the inner part. | He asserts that the dimness came on suddenly; as he was walking "a lot of gnats seemed to come before the sight;" he believes the sight has not got much worse since that time. | 1870, *June* 23. Vision 16 J.; still nothing in the distance. *Nov.* 24. The same. |

| Number. Date. Reference. | Name. Occupation. State of Health. | Age. Duration of Amaurosis. | Use of Tobacco and Alcohol. | State of Eyes. | Other Symptoms. | Progress and Remarks. |
|---|---|---|---|---|---|---|
| 19. 1870, May 26. F., p. 39. | William Doncaster. | 54. 12 months. | Has smoked about half an ounce daily for thirty-five years, *shag* tobacco. | Vision 20–100, and 16 J.; not improved by glasses. The ophthalmoscopic changes are not far advanced. The outer half of each disc is paler than the inner half; this is more marked in the right. | Gradual failure. He was very sick when he learnt to smoke; he has been to sea, and says that he has not been seasick. | 1870, *Sept.* 1. Vision 10 J. *Nov.* 7. Reads words of 4 J. without any glass. Still smokes a very little (half an ounce a month). |
| 20. 1870, Oct. 3. F., p. 71. | Fredk. C. Henderson, married, a clerk. In good health. | 30. 2 years. | Has smoked an ounce a week. Has never been intemperate. | Vision with the left eye (the worst), he cannot see one's hand. Pupils of normal size, but act very sluggishly. Discs are grey-white, especially at their outer halves. | Gradual failure; sight has been as bad as now for ten months. If he smoked more than his usual quantity it made him ill. | He left off smoking a year before he applied for advice. *Note* his inability to smoke more than a small quantity of tobacco. |
| 21. 1870, July 7. F., p. 67. | William Heatley. | 50. 15—18 months. | Has smoked half an ounce daily for thirty years, *cavendish* tobacco. | Vision 20-200, and 20 J. Discs somewhat pale, especially at the outer parts; left rather more so than right. There are slight iritic adhesions. | Gradual failure; no pain. | Had excellent sight formerly. |

| | | | | | | |
|---|---|---|---|---|---|---|
| 22. 1870, Oct. 17. F., p. 86. | John Liddle, joiner. | 35. 7 months. | Has smoked two ounces weekly for sixteen years, *shag and pig-tail* tobacco. Has drunk alcoholic liquors perhaps somewhat largely. | Vision, cannot count fingers at 3 feet. Discs atrophied; the left is more advanced than the right. | He asserts that his sight failed rather quickly, in two or three weeks, but without pain. He never could smoke very easily, and was always faint and sick if he smoked more than usual. | Lives a long way off, and cannot come again. |
| 23. 1870, Sept. 5. F., p. 89. | William Owen. | 63. 6 weeks. | Has smoked for more than twenty years, *shag* tobacco; latterly has used a quarter to half an ounce daily. | Vision 20–30, and 8 J.; not improved by glasses for distance. Discs decidedly paler at the outer than the inner halves; left, worse. | Gradual failure; complains of a fog. | For the last year has smoked more than he used to do; has had family troubles |
| 24. 1870. Nov. 14. F., p. 102. | Thos. Comery, bricklayer. In excellent health; dark hair and skin. | 28. 3 months. | Has smoked more or less for twenty-three years, largely for a year and a half, now a quarter of an ounce daily, *shag* tobacco. Drinks freely, and often gets drunk; drinks beer. | Vision 20 J.; cannot see 200 at 10 feet. Is hypermetropic. Discs very white, margins clear; arteries little, if at all, diminished. | He gets rather giddy at times, but is not otherwise ill. | Believes present failure of sight began suddenly after exposure to the sun on a very hot day. Sight has continued to get worse ever since. Had great difficulty in learning to smoke when a boy. |

| Number. Date. Reference. | Name. Occupation. State of Health. | Age. Duration of Amaurosis. | Use of Tobacco and Alcohol. | State of Eyes. | Other Symptoms. | Progress and Remarks. |
|---|---|---|---|---|---|---|
| 25. 1870, Nov. 24. F., p. 105. | Charles Hopkins, engine-driver. Married ; four children, all healthy. He is in good health, pale, dark hair. | 35. 6 months. | Has smoked half an ounce daily for eighteen years, *shag* tobacco. Drinks a very moderate amount of beer with his meals. | Vision 20–50, and 8 J. Pupils act fairly well, and are of normal size. Discs decidedly pale at the outer halves ; vessels not diminished. | Very gradual failure, has been nervous lately, but has not smoked more than usual. Had no special difficulty in learning to smoke. Has never been to sea. | Had very sharp sight formerly. |
| 26. 1870, Nov. 28. F., p. 109. | Israel Meyers, packing case maker. Fairly healthy-looking; head bald. Has had "rheumatism" in hand and foot ; was once laid up with it. | 39. 2 years. | Has smoked for eighteen years ; until a year ago he used half an ounce daily, since that time only half as much, *shag* tobacco. Has drunk beer and spirits moderately. | Vision, cannot count fingers ; the left is a little worse than the right. Pupils of medium size, motionless. Discs very white, sclerotic sheath plainly seen ; vessels not diminished. | Gradual failure ; has had no pain or inflammation in the eyes. Of late, whenever he has smoked more than usual, it has made him nervous and shaky. Had considerable difficulty in learning to smoke. | |

| | | | | | | |
|---|---|---|---|---|---|---|
| 27. 1870, Nov. 28. F., p. 112. | William Packer. | 40. 2½ years. | Has smoked for twenty-four years; never smoked largely, from half to three quarters of an ounce weekly, *shag* tobacco. Has drunk beer moderately. | Vision, can just tell light from darkness. Discs very white. | His sight began to fail in August, 1868; he believes that it began to fail suddenly on a hot day, and that it afterwards became worse. Learnt to smoke with difficulty, and has always been easily made sick and nervous by over-smoking. In boyhood he went to sea on board a collier, and was not troubled with sickness. | Has been under Mr. Couper's care for a long time; he continued to get worse after he left off smoking, and has been as bad as he is now for a year and a half. |
| 28. 1870, June. F., p. 117. | J. Lack, married, farm-laborer, | 42. 18 months. | Has smoked for twenty-five years; sometimes as much as half an ounce daily, *shag* tobacco. Of late years has drunk a good deal of beer (two or three pints daily). | Vision about 16 J.; nothing in the distance. Myopic refraction. Discs in condition of white atrophy, especially at outer halves. There are also crescents, with ill-defined margins, probably advancing. Not improved for distance by concave glasses. | Sight has failed gradually. He says he could see well *in the distance*, as well as for reading, until about a year and a half ago. His sight (he says) varies a good deal, and is best in the dusk. Smoking has never disagreed with him. | It seems probable that the myopia and the atrophy of discs have to some extent advanced together. He has never used his eyes much at close work. 1870, *Dec.* 1. Sight no worse, perhaps a little better; 16 J. Has not smoked at all. Is better in health. |

| Number. Date. Reference. | Name. Occupation. State of Health. | Age. Duration of Amaurosis. | Use of Tobacco and Alcohol. | State of Eyes. | Other Symptoms. | Progress and Remarks. |
|---|---|---|---|---|---|---|
| 29. 1870, Dec. 5. F., p. 125. | Wasdall Meadway, single. Has been farming, etc., at Cape and in Australia. In splendid health ; light hair. Was once ill, more or less, two years ago, with "liver complaint." | 36. 6 months. | Began to smoke twenty-five years ago ; has smoked quite regularly for eighteen years half an ounce daily ; smoked a sort of roll tobacco ("Boor tobacco") at the Cape. Drinks a fair quantity of beer, and some gin. | Vision 20–200, and 12 J.; not improved by glasses. Discs decidedly paler at outer third; vessels of good size. | Gradual failure. Learnt to smoke easily ; it never disagrees with him, or makes him nervous. Is a good sailor. | |

SUMMARY.—The five worst cases are, 20, 22, 24, 26, 27. In each the patient had been a *smoker for a long time* (average 19½ years, excluding No. 20, in which this item is not given. In several of them (excepting 24 and 26) the *quantity of tobacco smoked had been comparatively small.* The quantity of tobacco used by the two patients whose sight was worst of all (Nos. 20 and 27) was less than that used by any other patient. The *duration of the amblyopia* in these severe cases was (with the exception of No. 24) considerable, the average (excluding No. 24) being 21¼ months. There were *other signs of intolerance of tobacco* in the same five cases. Only one other case (No. 6) is noted as having suffered any other unpleasant symptoms, and these were apparently slight. In all the worst cases the patients had used alcoholic drinks, and two of them had been great drinkers.

In the *worst case of all* (No. 27) the patient had smoked very little compared with most of the others; he had had difficulty in learning to smoke, and smoking had often, in after-life, made him sick and nervous. He had smoked for 24 years. According to his statement, his sight continued to get worse after he left off smoking, but as he did not come under my care until his amaurosis was almost absolute, I cannot verify his assertions on this point.

